# Atypical septate junctions maintain the somatic enclosure around maturing spermatids and prevent premature sperm release in *Drosophila* testis

**DOI:** 10.1242/bio.036939

**Published:** 2019-01-11

**Authors:** Pankaj Dubey, Tushna Kapoor, Samir Gupta, Seema Shirolikar, Krishanu Ray

**Affiliations:** Department of Biological Sciences, Tata Institute of Fundamental Research, Mumbai 400005, India

**Keywords:** Septate junctions, Discs-large-1, Neurexin-IV, Spermiation, Somatic cyst cells, *Drosophila*

## Abstract

Tight junctions prevent paracellular flow and maintain cell polarity in an epithelium. These junctions are also required for maintaining the blood-testis barrier, which is essential for sperm differentiation. Septate junctions in insects are orthologous to the tight junctions. In *Drosophila* testis, major septate junction components co-localize at the interface of germline and somatic cells initially, and then condense between the two somatic cells in a cyst after germline meiosis. Their localization is extensively remodeled in subsequent stages. We find that characteristic septate junctions are formed between the somatic cyst cells at the elongated spermatid stage. Consistent with previous reports, knockdown of essential junctional components – Discs-large-1 and Neurexin-IV – during the early stages disrupted sperm differentiation beyond the spermatocyte stage. Knockdown of these proteins during the final stages of spermatid maturation caused premature release of spermatids inside the testes, resulting in partial loss of male fertility. These results indicate the importance of maintaining the integrity of the somatic enclosure during spermatid coiling and release in *Drosophila* testis. It also highlights the functional similarity with the tight junction proteins during mammalian spermatogenesis.

This article has an associated First Person interview with the first author of the paper.

## Abstract (precis)

**Summary:** Septate junctions seal the somatic enclosure around maturing spermatids in *Drosophila* testis. The junction integrity, maintained by Dlg1 and NrxIV, is essential for keeping the somatic enclosure intact until the mature spermatids are released.

## INTRODUCTION

Germ cell development requires an appropriate microenvironment. In the male germline, it is provided by the somatic-origin cells, *viz.*, the Sertoli cells in mammals and the somatic cyst cells (SCCs) in *Drosophila* (Griswold, 1998; Zoller and Schulz, 2012). Both these cell types insulate developing germ cells from body fluids and thus from the immune system. In mammalian testis, this isolation is accomplished by a specialized structure called the blood-testis barrier (BTB) (Cheng and Mruk, 2012). Tight junctions (TJ) form an essential part of the BTB (Mruk and Cheng, 2015). In an epithelium, TJs restrict the paracellular flow of solutes from the lumen, as well as separate the apical and basolateral domains of the plasma membrane (Hartsock and Nelson, 2008). In testis, TJs between Sertoli cells at the BTB play a significant role in maintaining the architecture of the seminiferous tubule, as well as the progression of spermatogenesis. The testis-specific knockout of Claudin-11 (Cldn11), an essential component of TJs in testis, leads to detachment of Sertoli cells from the basement membrane, thereby severely affecting the progression of spermatogenesis and the reproductive output (Mazaud-Guittot et al., 2010). Loss of another TJ protein, Zona-occludens-2 (ZO-2), from the Sertoli cells leads to mislocalization of a number of BTB proteins such as Cldn11, the gap junction protein Connexin-43 and actin, which leads to loss of BTB integrity, and a decrease in male fertility (Xu et al., 2009). Together, these observations suggest that TJs maintain the integrity of the seminiferous tubule and the BTB.

Septate junctions (SJs) in insects are considered to be the functional equivalent and evolutionary precursor to TJs (Banerjee et al., 2006). The Claudin family of proteins are essential for the formation these junctions and maintenance of the barrier function (Furuse et al., 1998a,b; Nelson et al., 2010; Wu et al., 2004). Some of the other components of SJs are Neuroglian (Nrg), Neurexin IV (NrxIV), Na^+^/K^+^-ATPase-α, Nervana (Nrv2), Lachesin (Lac), Discs-large-1 (Dlg1), Coracle (Cora) and Fasciclin III (Fas III) (Banerjee et al., 2006; Woods et al., 1996)*.* SJs play a critical role in developing tissue architecture and function. For instance, loss of *dlg1* disrupts the SJs and results in abnormal growth and fusion of imaginal discs (Woods and Bryant, 1989). Similarly, the loss of *cora* causes dorsal closure defects (Fehon et al., 1994), and the blood-nerve barrier fails to form in *nrx* homozygous mutants due to the disruption of SJs (Baumgartner et al., 1996). Independent of their role in sustaining the barrier function, N^+^/K^+^ ATPase-α and nervana-2 are also known to be involved in controlling the size of the tracheal tube (Paul et al., 2003). These observations suggest that apart from their classical role of forming a diffusion barrier, SJs are also involved in cell signaling and maintenance of epithelial integrity.

In *Drosophila* testis, spermatogonia develop inside an enclosure formed by two somatic-origin cyst cells (SCCs) that undergo extensive morphogenesis and ultimately differentiate into the head (HCC) and tail (TCC) cyst cells during spermatid elongation (Lindsley and Tokuyasu, 1980; White-Cooper, 2004). Each spermatid elongates to ∼1.8 mm after meiosis inside the somatic cyst enclosure. Subsequently, they are individualized, coiled and released into the seminal vesicle (SV) as mature sperm (Lindsley and Tokuyasu, 1980). The spermatogonial cysts become impermeable to the soluble dye, FITC-dextran, from an early stage of development (Fairchild et al., 2015; Gupta et al., 2018). Further, knockdown of NrxIV and Cora in SCCs permeabilizes the cysts and affects the spermatogonia to spermatocyte transition (Fairchild et al., 2015). Based on this evidence, SJs are suggested to form the permeability barrier from an early stage of spermatogenesis, which is critical for sperm development. However, transmission electron microscopy (TEM) study of *Drosophila* testis reported classical SJs between the SCCs during the post elongation stages (Tokuyasu et al., 1972). Hence, it was unclear whether the SJ proteins form the fluid barrier without establishing typical junctions. Also, the role of SJs at the later stages was unclear.

Here, we report a new role of SJs during the final stages of spermatogenesis. We found that several components of SJs localized at the interface of SCCs and germline cells from the spermatogonial stages, which subsequently reorganized at the somatic cell interface during spermatid elongation. Consistent with an earlier prediction (Tokuyasu et al., 1972), we noted that the junction marked by the SJ proteins migrates towards the caudal end of the enclosed spermatid head bundle after individualization. TEM analysis also suggested that typical SJs form between the SCCs after spermatid individualization. The loss of Dlg1 in SCCs during the spermatid coiling and maturation stage disrupted the localization of other SJ components at the HCC–TCC interface and resulted in the premature release of spermatids inside the testis. Time-lapse imaging further indicated that the spermatids are likely to be released during the cyst rotation in the terminal epithelium (TE) region at the base of the testis in the *dlg1* RNAi background. Altogether, these observations validated that the SJs between HCC and TCC form after the spermatid individualization and further suggested that the junction is required to maintain the mechanical integrity of the somatic cyst enclosure during its migration through TE, preceding sperm release.

## RESULTS

### Morphogenesis of the cellular interfaces marked by SJ proteins during sperm development

**Fig. 1.**
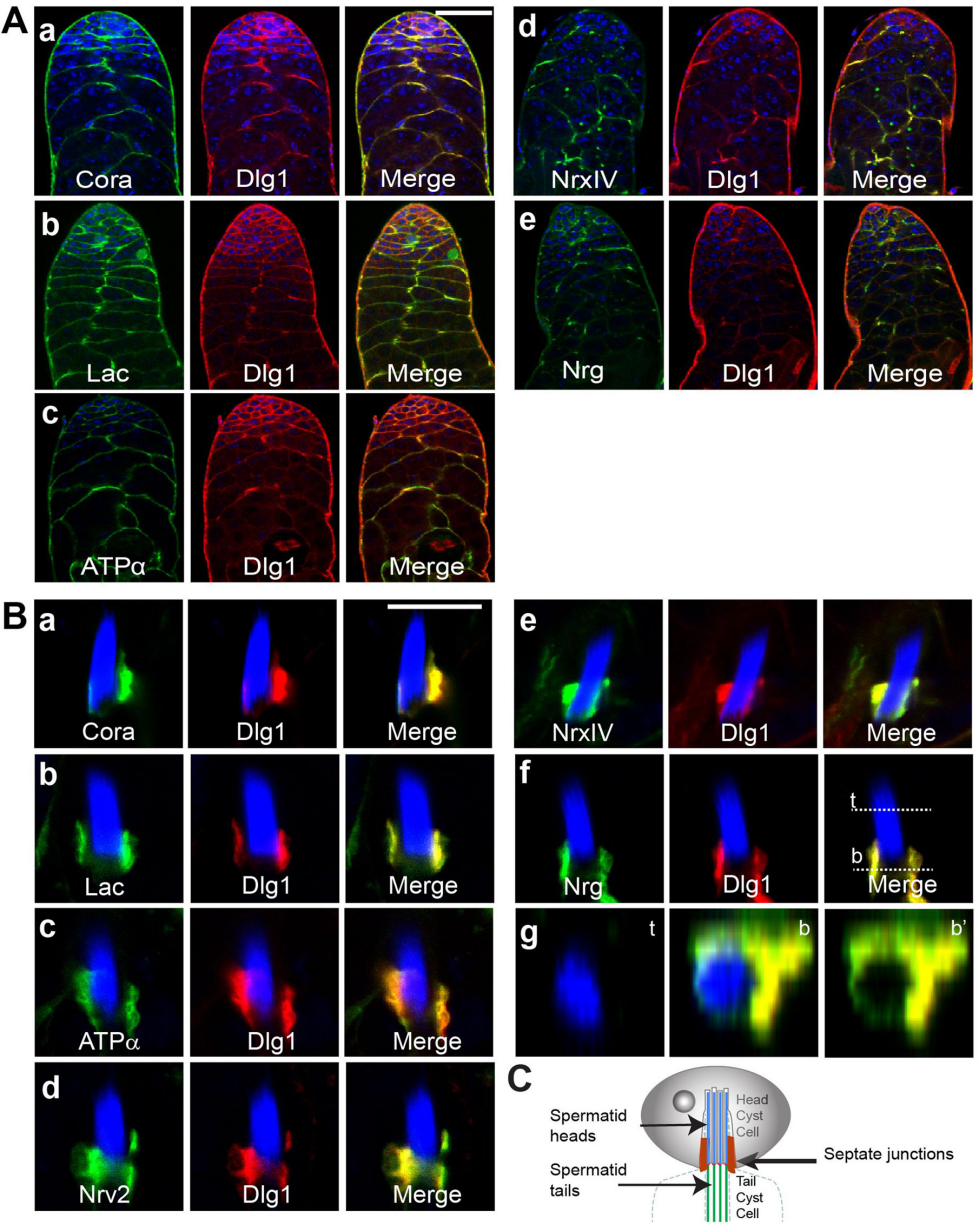
**Major components of SJs co-localize during early and late stages of spermatogenesis.** (A) Apical tips of testes showing co-localization of the SJ proteins – Lac-GFP (b), ATPα-GFP (c), NrxIV-GFP (d) and Nrg-GFP (e) – with anti-Dlg1 (red) immunostaining at the interface of the germline and somatic cells. Dlg1-GFP testes (red) were immunostained with anti-Cora (green; a). All specimens were stained with Hoechst dye (blue) marking the nuclei. Scale bar: 50 µm. (B) The SJ proteins also localize caudal to the compact nuclei bundle (NB) of the mature spermatids during the late stages. Hoechst staining, marking all nuclei, is in blue. (g) The X-Z digital section through the top (t) and bottom (b) parts of the specimen shown in panel f. It indicates that the SJ proteins localize all around the NB. Scale bar: 10 µm. (C) Schematic describes the position of the junction between the head and tail cyst cells.

To observe the localization and developmental reorganization of SJs at the cellular interfaces during spermatogenesis, we carried out a limited screen using protein trap lines and antibody staining of adult testes. We identified Nrg, NrxIV, Na^+^/K^+^-ATPase-α, Nrv2, and Lac enrichment at the soma–germline interface by using protein trap lines (green, Fig. 1Ab–e). Anti-Dlg1 immunostaining (red, Fig. 1Ab–e) (Parnas et al., 2001) suggested that all the above SJ components co-localize with Dlg1 at all stages. Further, the pattern matched with that of the endogenous Dlg1-GFP enrichment (red, Fig. 1Aa,Ba) and anti-Cora immunostaining (green, Fig. 1Aa,Ba) (Fairchild et al., 2015). The SJ components localized at the germ–soma interface until the completion of meiosis. Subsequently, we noticed a condensed and prominent localization near the caudal end of the spermatid head bundles during the coiled stages at the testis base (Fig. 1B,C). Together, these results indicated that the cellular interface marked by these proteins undergoes extensive reorganization between the early and late stages of spermatogenesis.

**Fig. 2.**
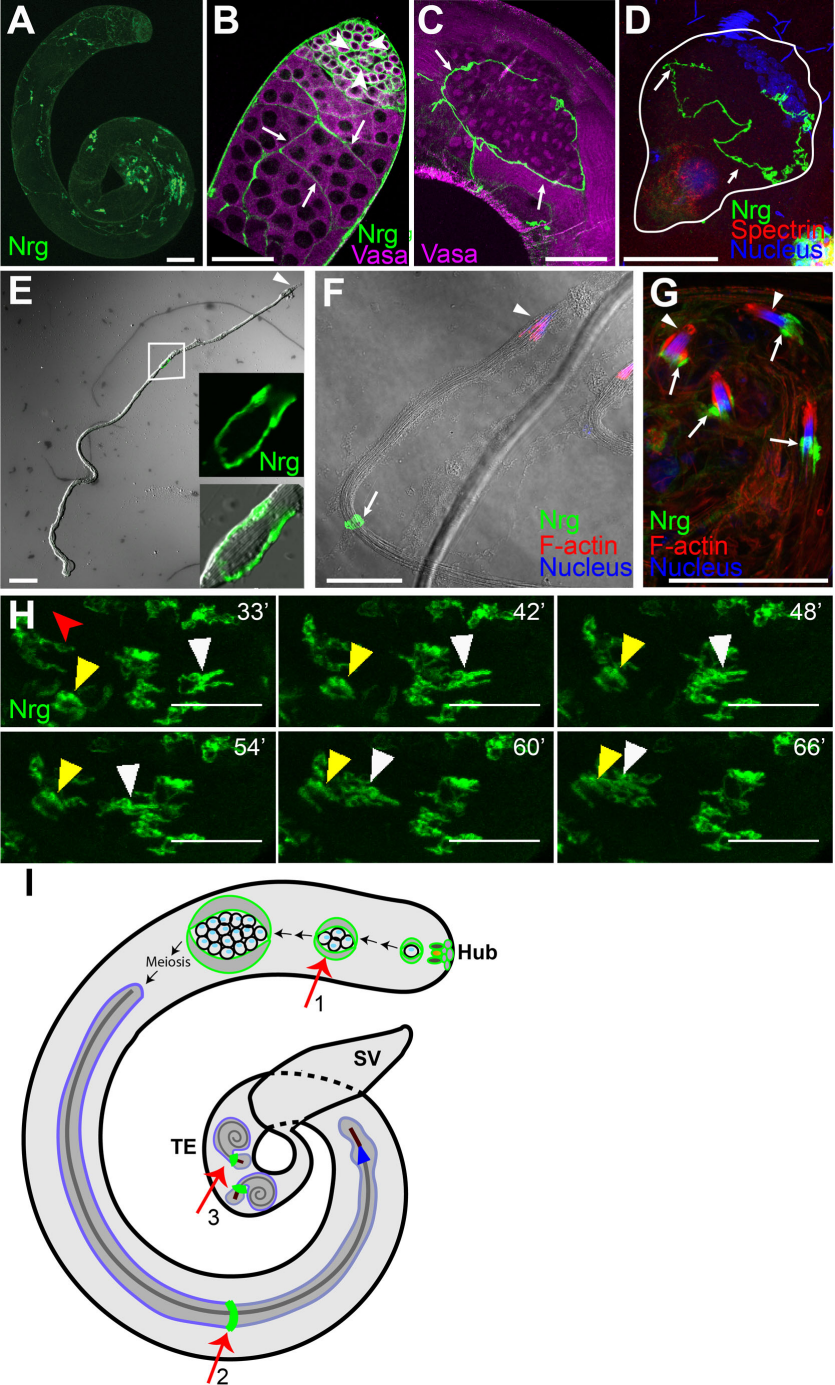
**Morphogenesis of the SJ protein Nrg during spermatogenesis.** (A–C) Nrg-GFP (green) expressing testes stained with the anti-Vasa antibody (magenta). (A) Low magnification image of Nrg-GFP testis shows the presence of Nrg at different stages. (B) Apical end of the testis shows Nrg-GFP localization around individual spermatogonia (arrowheads) at the initial stage. It is then restricted to the cyst perimeter (arrows) of the primary spermatocyte stages. (C) A post-meiotic cyst shows the presence of Nrg-GFP along the cyst perimeter. Nrg-GFP is excluded from the germ cell perimeter inside the cyst enclosure from the spermatocyte stage onwards. (D–F) Squash preparation: Nrg-GFP (green) testes immunostained with the anti-Spectrin antibody (red, D), Hoechst dye (blue) and Phalloidin (red, F). (D) An early elongating cyst (outlined by white boundary) shows polarization of the spermatid nuclei (blue) and tails (red), and localization of Nrg-GFP (arrows) at the HCC–TCC interface. (E–F) Elongated spermatid cysts from Nrg-GFP testes were isolated and stained for the IC (red) and nucleus (blue), present at the rostral ends of the cyst (arrowheads). The HCC and TCC are highly extended at this stage, and a condensed form of Nrg-GFP (arrows) between these two cells was seen in the middle region. (G) Coiled stage spermatids from Nrg-GFP testes, stained for F-actin (marking the actin cap, red) and nucleus (blue). The arrows indicate localization of Nrg caudal to the NB (arrowheads, blue). Note that the position of the Nrg-GFP has changed post-individualization. (H) Time-lapse images of Nrg-GFP testis show the movement of the Nrg-GFP structure (yellow and white arrowheads) towards the basal end of the testis. The red arrowhead indicates the direction of the SV. Scale bars: 50 µm. (I) Schematic illustrates the morphogenesis of domains marked by SJ proteins in adult testis. The SJ protein is marked in green, spermatid tail in grey, spermatid nuclei in maroon and IC in blue. For simplicity, only one spermatid is shown within a cyst enclosure. The arrows indicate the position of SJs proteins during the spermatogonial stage (1), elongated stage (2) and coiled stage (3). Schematic is not to scale.

Next, we monitored the enrichment of SJ components at the SCC interfaces using endogenous Nrg-GFP protein-trap localization (*Nrg-GFP^PT^*) (Fig. 2A). Nrg-GFP was found enriched around all germ cells, identified by anti-Vasa immunostaining (Renault, 2012), and SCCs at the early spermatogonial stages (arrowheads, Fig. 2B). This is consistent with a previous report, which analyzed the localization of Dlg1 (Papagiannouli and Mechler, 2009). Subsequently, it relocalized at the cyst boundary during the late spermatogonial stage (arrows, Fig. 2B) and post-meiotic spermatocyte stage (arrows, Fig. 2C), indicating a gross reorganization of the junctions during these stages. The elongating stage cyst can be identified by the polarized arrangement of 64 spermatid nuclei on one side and Spectrin caps on the other (Ghosh-Roy et al., 2004). Squash preparation of testis revealed localization of Nrg-GFP at the middle of the early elongating (Fig. 2D), as well as the fully elongated cysts (Fig. 2E). Consistent with an earlier report (Fairchild et al., 2015), these observations suggested that SJ proteins localize at the HCC–TCC boundary from the elongation stages onwards.

NBs of elongated spermatids are positioned near the testis base. The compact localization of Nrg-GFP around the middle of the cyst (arrow, Fig. 2F) persisted until the beginning of the individualization stage [identified by the presence of F-actin rich individualization complexes (IC) caudal to the spermatid nuclei] (arrowhead, Fig. 2F). Subsequently, the spermatid tails coil up after individualization. Post individualization, Nrg-GFP, along with several other SJ proteins, localized near the caudal end of the compacted nuclei bundle (NB) of the spermatids (arrows, Fig. 2G; Fig. 1B). Thus, the junction appeared to move towards the NB during individualization or coiling stages of spermatogenesis. Time-lapse imaging in the mid-region of testis further revealed that occasionally some SJs moved towards the base (yellow and white arrowheads, Fig. 2H; Movie 1). The SJs are likely to form between the HCC and TCC. Therefore, the movement of SJs towards the base may suggest a reorganization of the HCC and TCC morphology (Fig. 2I), either before or during coiling, which is consistent with the model proposed earlier (Tokuyasu et al., 1972).

### SJs were first observed between the somatic cyst cells during the elongated spermatid stage

**Fig. 3.**
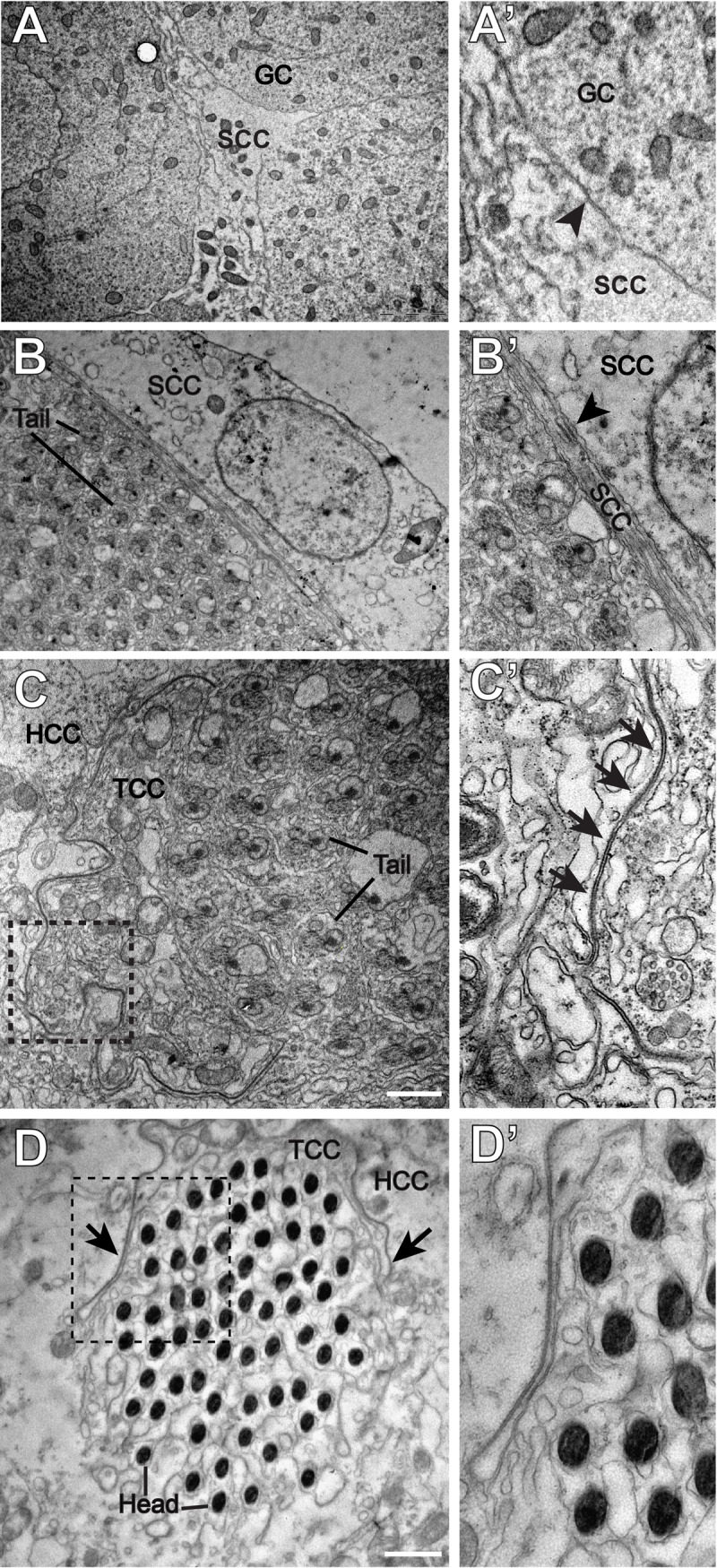
**Ultrastructural analysis of the germ–soma and soma–soma interfaces in adult testis.** (A) Electron micrograph of the spermatogonial stages shows a somatic cyst cell (SCC) and germ cells (GCs). The junction between the SCC and GC is seen (arrowhead, A′). (B) Section through an elongated cyst, as can be identified by the spermatid tails with major and minor mitochondria, along with the associated SCC. Note that the junction between the SCCs does not resemble an SJ (arrowhead, B′). (C) Electron micrograph through the tails of a more mature, pre-individualized cyst shows the presence of a septa-like pattern between the two surrounding cyst cells. C′ shows the magnified image of the boxed region in C. Arrows indicate a ladder-like SJ between the plasma membranes of the two SCCs. (D) Section through the spermatid heads at the coiled stages, with surrounding HCC and TCC. D′ shows a magnified image of the boxed region in D. Ladder-like arrangement of SJs between the two cyst cells can be seen around the sperm head. Note that similar to the results obtained by confocal microscopy, the junction was relocated just caudal to the sperm heads. Scale bars: 1 µM.

Structurally, SJs are classified into two types – pleated and smooth. Pleated SJs (pSJs) are found in ectoderm-derived epithelia. The pSJs have a typical ladder-like arrangement of electron-dense elements at ∼15 nm-wide intervals along the membrane interface between two epithelial cells (Banerjee et al., 2006; Locke, 1965), while smooth SJs have a parallel arrangement (Banerjee et al., 2006). To identify the type of junction formed by the enrichment of the SJ proteins around the germline cells during the early spermatogonial stages, we examined the cellular interfaces within cysts in wild-type testis using TEM. It did not reveal the characteristic electron-dense structures typical of SJs at the interfaces between germline and somatic cells in the pre-meiotic cysts (Fig. 3A,A′). Some electron-dense structures were seen between the SCCs around the post-meiotic, elongated spermatids (arrow, Fig. 3B,B′). More prominent electron dense patterns resembling pSJs were found around the tails of fully elongated spermatids containing the major and minor mitochondrial derivatives (arrows, Fig. 3C,C′). These electron-dense SJs were also found near the nuclei of compacted spermatid head bundles at the base of the testis, which is characteristic of the post-individualized stages (arrow, Fig. 3D,D′). Together with the previous results (Tokuyasu et al., 1972), these observations further suggested that SJs are formed between the HCC and TCC during spermatid elongation, and maintained in subsequent stages.

### Knockdown of Dlg1 and NrxIV in the somatic cyst cells at an early stage arrested post-meiotic differentiation

A previous study in adult *Drosophila* testis reported that the expression of Cora and NrxIV in the SCCs is essential for forming a functional germ-soma permeability barrier during the spermatogonial stage, which is necessary for further germline differentiation (Fairchild et al., 2015). A subsequent report, however, showed that disruption of the permeability barrier by independent means did not always affect the spermatogonial division and differentiation (Gupta et al., 2018). However, as discussed earlier, SJ proteins also have roles other than serving as a diffusion barrier. For instance, a significant number of pole cells in *dlg1* homozygous mutant embryos fail to reach the gonadal pockets. Further, the male-specific mesoderm cells expressing Sox100B fail to get incorporated into the male gonad in stage 15 embryos (Papagiannouli, 2013). Loss of Dlg1 also affected spermatocyte differentiation in *Drosophila* larvae. It reduced the eyes-absent (Eya)-positive SCCs and induced germ cell death in the 16-cell spermatocyte cysts, indicating a role of Dlg1 in somatic differentiation as well as cyst survival (Papagiannouli and Mechler, 2009). Although *tj-Gal4* mediated knockdown of Dlg1 in SCCs during the spermatogonial stages disrupted the cyst permeability barrier and arrested differentiation, it did not affect the transit amplifying divisions of the spermatogonia (Gupta et al., 2018). Hence, we conjectured that in addition to maintaining the barrier function, the somatic Dlg1 activity might specifically regulate the transition to the meiotic stages in the male germline.

**Fig. 4.**
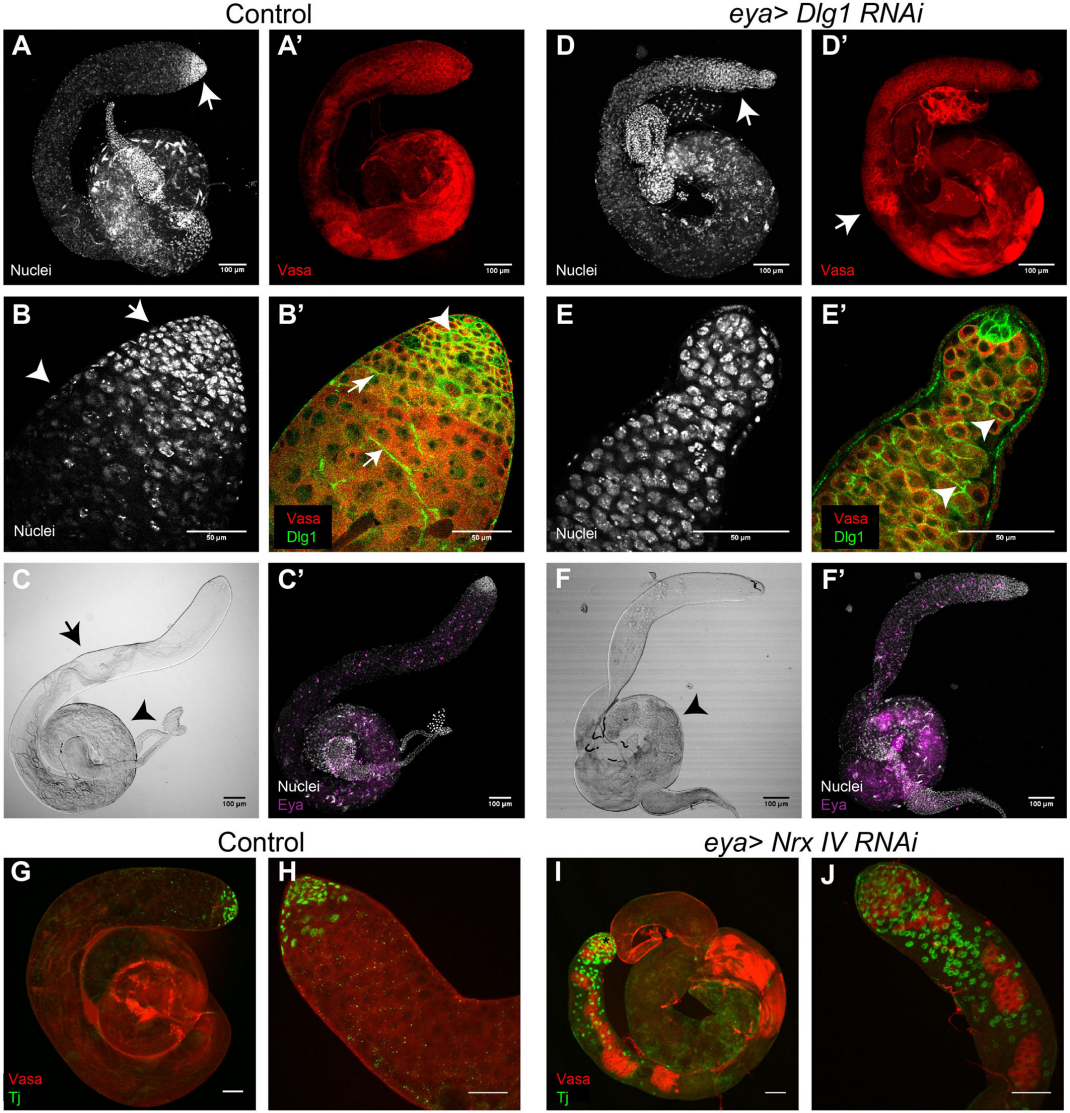
**Knockdown of Dlg1 and NrxIV during spermatogonial stages leads to a defect in proliferation and differentiation.** (A–B′) The control *eya>dsGFP* (*eya-Gal4/+; UAS-dsGFP/+*) testis stained with the Hoechst dye (white), anti-Dlg1 (green) and anti-Vasa (red) antibodies. (A) Hoechst staining shows tightly packed, condensed nuclei at the apical tip (arrow). (A′) Vasa pattern in control testis. (B) High magnification image of the apical tip shown in A. Arrow marks the condensed nuclear staining of mitotically active cells, while arrowhead marks the transition to meiotic stages, as indicated by comparatively less intense nuclear staining. (B′) Dlg 1 (green) localizes around the germ cells (arrowhead) as indicated by Vasa (red) initially, and then on membranes of the SSCs (arrows). (*n*=13). (C) DIC image indicates the presence of elongated/individualized cysts (arrow), as well as coiled cysts (arrowhead). (C′) Control testis shows the distribution of Eya (magenta) positive somatic cyst cells. (D–E′) *eya>dsDlg1* (*eya-Gal4/UAS-dsDlg1*) testis stained with the Hoechst dye (white), anti-Dlg1 (green) and anti-Vasa antibodies (red). (D) Brightly stained spermatogonial nuclei are extended to the middle region of the testis. (D′) Pockets of Vasa staining, usually restricted more apically, extend until the middle region of the testes (arrow). (E) High magnification image of the apical tip of the testis shown in D. Note that the apical tip is shrunken as compared to control testes in B. (E′) Arrowheads indicate anti-Dlg1 (green) immunostaining around the germ cells. There is no somatic Dlg1 immunostaining. (*n*=13). (F) DIC image indicates a lack of elongated/individualized cysts and a decrease in the density of coiled cysts (arrowhead). (F′) Distribution of Eya (magenta) positive somatic cyst cells in *eya>dsDlg1* testis. Distribution is similar to control in C′. (G–H) Control (CantonS) testis stained with anti-Vasa (red) and anti-Tj (green) antibodies. Note that the Tj-expressing somatic cells are restricted near the apical tip of the testis (H). (*n*=10). (I–J) *eya>dsNrxIV* (*eya-Gal4/UAS-dsNrxIV*) testis stained with anti-Vasa (red) and anti-Tj (green) antibodies. Patchy expression of Vasa indicates defects in germline differentiation. Tj expression is expanded. Also note that the testis appeared shrunken, similar to what was seen upon the knockdown of Dlg1. (*n*=10). Scale bars: 50 µm, unless specified otherwise on the image.

Therefore, to understand the role of the SJs during the spermatogonia to spermatocyte transition, we knocked down two essential components of the junction – Dlg1 and NrxIV – using *eya-Gal4*, which is expressed in both the SCCs from the 4-cell spermatogonial stage onwards (Fig. S1A,A′) (Fabrizio et al., 2003; Leatherman and Di Nardo, 2008). The *eya-Gal4>UAS-dsGFP* (*eya>dsGFP*) testis contained tightly packed, mitotically-active, spermatogonial cells with condensed chromatin at the apex (arrows, Fig. 4A,B). The chromatin is de-condensed at the subsequent spermatocyte stages (arrowheads, Fig. 4B). In the *eya>dsDlg1* testes, the apical ends of testes were shrunken (Fig. 4D,E), testes were mostly filled with germ cells having compact chromatin morphology (Fig. 4D,E), and the anti-Dlg1 immunostaining was limited to the interface of the germline cells (arrowheads, Fig. 4E′). These testes had very few elongated and coiled spermatids, as compared to the control (Fig. 4C,F), indicating defects in the subsequent differentiation process. A similar differentiation defect was reported earlier in the NrxIV RNAi background (Fairchild et al., 2015).

Germ cells of a spermatogonial cyst remain interlinked through inter-cellular bridges called ring canals, which are associated with membrane-rich structures known as fusomes. These structures are further branched with each division interconnecting all the germline cells within a cyst (Lu et al., 2017). The presence of a branched fusome is, therefore, considered a mark of spermatogonial differentiation and indicates syncytium amongst the germline cells. To test the effects of the loss of Dlg1 in the SCCs on fusome structure, we stained the control and mutant testes with anti-Hu-li tai shao (anti-Hts) (Terry et al., 2006), as well as the proliferation marker anti-phospho-histone 3 (pH3) (Gupta et al., 2018). The staining pattern revealed that Dlg1 knockdown does not disrupt the fusome morphology in the spermatogonial cysts (Fig. S2).

Although the anti-Eya immunostaining (Sheng et al., 2009) appeared in the SCCs at the appropriate region of the testis (Fig. 4C′,F′), immunostaining with another somatic marker anti-Traffic-jam (Tj) (Li et al., 2003), which is expressed in the early population of somatic cyst cells (Fig. 4G,H) (Hudson et al., 2013; Li et al., 2003), revealed abnormal expansion of Tj-positive SCCs in the NrxIV knockdown testes (Fig. 4I,J). Together, these results reaffirmed that loss of Dlg1 and NrxIV from the SCCs during the mitotic–meiotic transition blocks cyst differentiation and leads to eventual loss of germ cells beyond the early spermatogonial stages.

### Knockdown of SJs during the coiling stages disrupted spermatid bundles

**Fig. 5.**
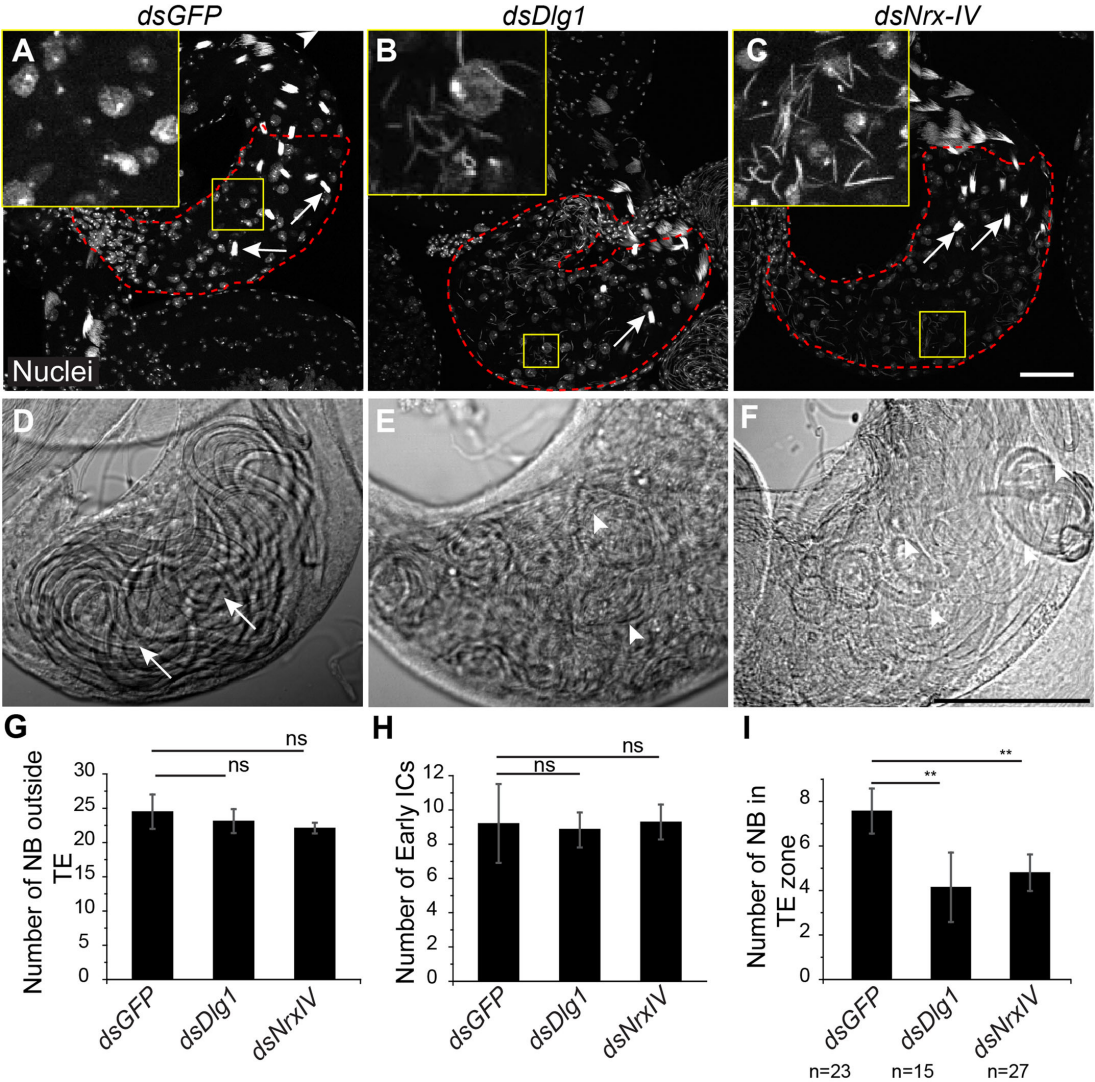
**Knockdown of SJ components in the cyst cells during late stages affects NB integrity and spermatid coiling.** (A) Control *PpY>dsGFP* (*PpY-Gal4/UAS-dsGFP*) testes stained with Hoechst (white) to mark the nuclei. Arrows mark the NBs in the TE region (red dashed outline). The inset shows a high magnification image of the testis base. (B,C) *PpY>dsDlg1* (*UAS-dsDlg1/+; PpY-Gal4/+*) and *PpY>dsNrxIV* (*UAS-dsNrxIV/+; PpY-Gal4/+*) testes stained with the Hoechst dye. Comparatively fewer intact NBs (arrows) can be seen in the TE zone (red dashed outline), whereas an unusually large number of individual spermatid heads were found inside these testes (insets). (D–F) Bright-field images show the basal end of control (D), *PpY*>*dsDlg1* (E) and *PpY*>*dsNrxIV* (F) testes. The arrows indicate intact coiled tail bundles in D, while arrowheads point towards the disrupted tail bundles in E and F. (G–I) Histograms indicate the average intact NBs outside TE (G), the number of early ICs (H) and the number of NBs inside the TE (I) in control (*n*=23), *PpY>dsDlg1* (*n*=15) and *PpY>dsNrxIV* (*n*=27) testes. *P*-value (**<0.01) was calculated using the Mann–Whitney *U*-test. ns, not significant; scale bars: 50 µm.

To determine the role of SJs in the post-meiotic stages, we used *PpY-Gal4*, which expresses in the SCCs after meiosis (arrowhead, Fig. S1B,B′) (Jung et al., 2007). The expression of *PpY>dsDlg1* selectively abolished Dlg1 immunostaining from the HCC–TCC interface in cysts in the TE region (Fig. S3E), suggesting Dlg1 is effectively knocked down at the terminal stages by dsRNA expression. Knockdown of Dlg1 also eliminated Cora immunostaining from around NBs in the TE region (Fig. S3F). These results suggested that SJs may be disrupted during the terminal stage of spermiation in the *PpY>dsDlg1* background. We also found an unusually large number of free spermatid heads and a concomitant decrease in intact NBs in the TE region (insets, Fig. 5A–C,I) The corresponding bright field images revealed improperly coiled spermatid tails (Fig. 5D–F). In comparison, the number of total bundles outside the TE and the early individualizing bundles were not affected (Fig. 5G,H). The morphology of the early and progressed ICs was also normal in these testes (Fig. S4). Therefore, these observations suggested that the SJ proteins are required in SCCs to keep spermatids tightly bundled and coiled during the final stages of spermiation, and prevent abnormal release.

### Knockdown of Dlg1 in SCCs during spermatid coiling induced the premature release of spermatids within the testis

The cyst enters the TE region at the testis base with the NB of coiled spermatids facing towards the SV. Subsequently, the cyst rotates after entering the TE, and the NB of coiled spermatids now orient away from the SV at the time of release. Time-lapse imaging also shows that the spermatids are pulled back from the HCC with their tails leading through the testicular duct during the release (Dubey et al., 2016). Thus, the cyst rotation is suggested to facilitate the release by bringing the tail bundles closer to the testicular duct. The SJs between HCC and TCC remain intact during the release process (Dubey et al., 2016). We conjectured that the HCC–TCC interface would be subjected to a high level of tension during the spermatid coiling, and subsequent cyst movement and rotation through the TE. During this process, SJs at the HCC–TCC interface could impart mechanical stability, balancing the tension at the interface and preventing the abnormal release.

**Fig. 6.**
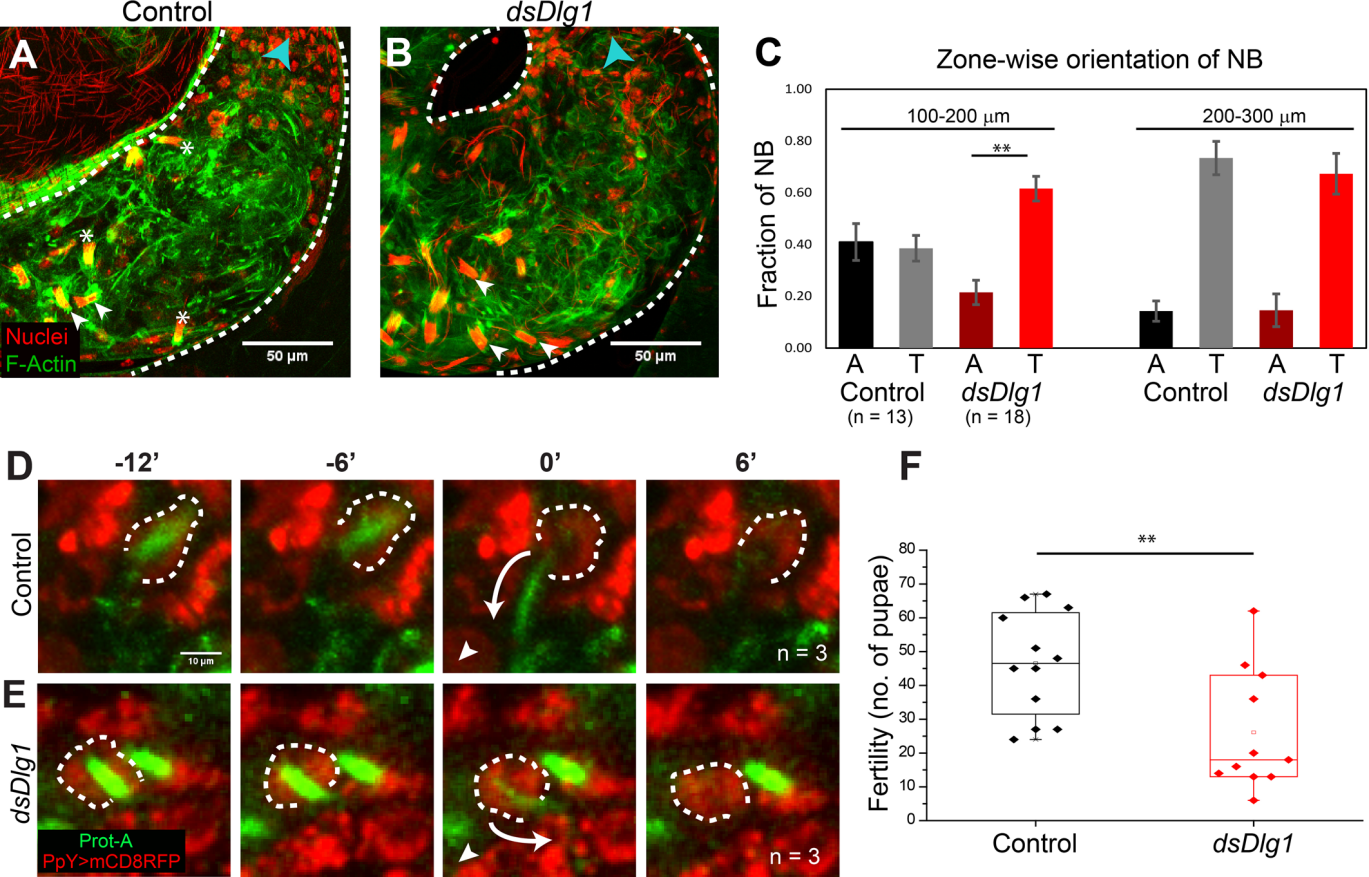
**Dlg1 loss from the HCC–TCC interface at the coiling stage causes premature sperm release inside the testis.** (A,B) NB orientations in the control *PpY>dsGFP* (*n*=13) (A) and the *PpY>dsDlg1* (*n*=18) (B) testes. Testes were stained with the Hoechst dye (red) and Phalloidin (green), and the position of the actin cap was used as an indicator of whether the NBs were facing towards (white arrowhead) or away (asterisk) from the SV (direction of SV marked by blue arrowhead). Note the decrease in bundles facing away from SV in 100–200 µm region in B. (C) Zone-wise distribution and the orientation of NBs in the TE region. Distances from the SV were measured from the proximal end of the testicular duct. ‘A’ denotes the NB orientation away from SV, and ‘T’ denotes orientations towards the SV. *P*-value (**<0.01) was calculated using the Mann–Whitney *U*-test. Apart from these two classes, a fraction of NBs were found with intermediate orientations that are not represented on the graph. Spermatids exit testis in the ‘A’ orientation, as shown by Dubey et al. (2016). (D,E) Time series from live imaging of the testes. Protamine A (green) marks spermatid head, while mCD8-RFP (red) marks cyst cell (dashed outline). The white arrowhead in each panel indicates the direction of SV and the curved arrow marks the NB retraction trajectory. In control *(ProtA-GFP/UAS-Dicer; PpY-Gal4>UAS-mCD8-RFP/+)* testis (D), the spermatid heads retracted (white arrow at time 0′) in the direction of the SV. In the *dlg1* RNAi background (*ProtA-GFP/UAS-dsDlg1; PpY-Gal4>UAS-mCD8-RFP;+*) (E), spermatid heads retracted away from the SV (white arrow at time 0′), even though the cyst has not turned to face away from the SV. (F) Box plots depict the number of pupae produced by individual *PpY>dsGFP* (control) and *PpY>dsDlg1* males in 24 h. *P*-value (**<0.01) was calculated using the Mann–Whitney *U*-test.

To test this hypothesis, we checked the position-specific orientations of NBs in the TE region in the *dlg1* RNAi background. In control testes, NBs found at the 100–200 µm distance from the SV were oriented with equal propensity both toward (arrowheads, Fig. 6A) and away (stars, Fig. 6A) from the SV (black and grey bars, Fig. 6C). In comparison, a majority of the NBs in the 200–300 µm zone were found oriented towards the SV (Fig. 6C). In the *dlg1* RNAi background, a significant fraction of the relatively fewer NBs found in the 100–200 µm zone remained oriented towards the SV (arrowheads, Fig. 6B; dark and bright red bars, Fig. 6C). The distribution in the more distal zone (200–300 µm from SV) was similar to the control (Fig. 6C). In addition, we observed an abnormal accumulation of free spermatid heads which were scattered at the testis base. Together, these observations suggested that loss of Dlg1 in the SCCs disrupted NBs during cyst rotation through the TE before sperm release.

To confirm this, we captured time-lapse images from whole testis *ex-vivo*. The spermatid heads were labeled with *ProtamineA-GFP* and cyst cell membrane was labeled with *PpY>mCD8-RFP* in both the control (*ProtA-GFP/UAS-Dicer; PpY-Gal4>UAS-mCD8-RFP/+*) and *dlg1* RNAi (*ProtA-GFP/UAS-dsDlg1; PpY-Gal4>UAS-mCD8-RFP/+*) backgrounds. In the control testes, the NBs always retracted from the HCC during release in the direction of the SV (*n*=3, Fig. 6D; Movie 2). In the *dlg1* RNAi background, the spermatid heads retracted even though they were not facing away from the SV (*n*=3, Fig. 6E; Movie 3). As a result, they were released prematurely inside the TE region. Hence, we conclude that the loss of Dlg1 in SCCs leads to loss of SJs between the HCC and TCC, and that the integrity of this junction is critical to prevent cyst disruption and premature sperm release within the testis.

Finally, to understand the implications of the mechanical stability of the cyst enclosure during spermiation on male reproductive fitness, we carried out a fertility test. Individual *PpY>dsGFP* (control) and *PpY>dsDlg1* males were allowed to mate with three wild-type virgin females in separate vials, and the females were allowed to lay for 24 h (refer to the Materials and Methods). Subsequently, the number of pupae were counted in each vial. The results indicated that fertility of the *dlg1* RNAi males was significantly lower as compared to the control (Fig. 6F). Together with the observation of the time-lapse images, this result suggests that the premature release is detrimental to the male reproductive fitness.

## DISCUSSION

### SJs between the somatic cyst cells are remodelled during spermatid maturation

The SCCs forming the cyst capsule undergo extensive changes in cell shape and size during sperm development. The cyst enclosure is required for proper differentiation and maturation of the germ cells from the spermatogonia to coiled spermatid stages. It is intriguing how the cyst manages to keep the enclosure intact throughout spermatogenesis, withstanding considerable physical stress. In this study, we provide a systematic description of the septate junction morphogenesis during sperm development. Using Nrg-GFP as a marker of SJs and the TEM study, we show that though the SJ proteins are localized on both the germ cells and somatic cell membrane during the initial spermatogonial stage, the SJs between the SCCs are formed during spermatid elongation and are dynamically reorganized towards the later stages before spermiation. The condensation of the SJ proteins along the SCC interface during spermatid elongation may indicate that SCCs further differentiate acquiring cell polarity during this stage.

A similar reorganization of SJ proteins has been described during *Drosophila* embryogenesis. It was shown that in epithelial cells of the trachea, until stage 13, SJ components localize all along the basolateral edges, and by stage 15 they are localized exclusively to the apico-lateral domain (Tiklová et al., 2010). Similarly, other studies have shown that until stage 15 Cora localizes all along the basolateral domain of the cells of the salivary gland, and post-stage 15 they localize to the apico-lateral domain (Hall and Ward, 2016). Permeability tests further show that the occluding property of SJs is attained by stage 15, after their condensation at the apico-lateral domain (Paul et al., 2003), which is correlated to the formation of mature SJs around stage 16–17 as observed by ultrastructural studies (Tepass and Hartenstein, 1994). Together these observations show that the compact localization of SJ components at the apico-lateral domain during the differentiation of polarized epithelium marks the formation of mature and functional occluding junctions.

In the testis, the SJ proteins are initially localized on both the germline and somatic cell membrane during the spermatogonial stages, and the somatic permeability barrier is established from the 4-cell stage. Loss of the SJ components Dlg1, NrxIV and Cora from the germ–soma interface during this period distrupts the permeability barrier and affects subsequent differentiation to the spermatocyte stage (Fairchild et al., 2015; Gupta et al., 2018). A similar loss of permeability due to the knockdown of Armadillo/β-catenin, however, does not appear to affect the immediate differentiation to the spermatocyte stage (Gupta et al., 2018). Therefore, the SJ proteins Dlg1, Cora and NrxIV are likely to regulate the germline differentiation independent of their role in establishing the barrier function. Dlg1 is a well-characterized tumor-suppressor gene, loss of which leads to defects in proliferation and differentiation in multiple cell types. In the optic lobes and wing imaginal discs of *Drosophila,* the loss of Dlg1 leads to overgrowth phenotypes (Woods and Bryant, 1989). In the *Drosophila* ovary, Dlg1 is required in the follicle cells for their specification into posterior follicle cells (Li et al., 2009). In *dlg1/ZO-1* mutant mice, there is increased cell proliferation in the eye lens (Nguyen et al., 2003). Thus, studies in other contexts also suggest a role of Dlg1 in the regulation of cell proliferation and differentiation.

### Atypical SJs forms only after meiosis and during spermatid elongation

In concurrence with a previous report (Tokuyasu et al., 1972), the TEM data also suggests that a ladder-like, septate pattern is formed after the elongation stages, and we did not find the presence of SJs during mitotic and meiotic stages. In the mammalian testis, the BTB is formed after the mitotic stages and creates an isolated microenvironment for the meiotic and post-meiotic germ cells (Mruk and Cheng, 2015). We could not find any SJ-like feature between the germ cells or at the germ–soma interface during the early stages in TEM. The first electron-dense material appeared at the interface of the SCCs encapsulating elongated spermatids. Therefore, SJs are unlikely to contribute to establishing the permeability barrier during the spermatogonial stages.

After the reorganization, SJ proteins localize at the HCC–TCC interface, which is further compacted during spermatid individualization. Classically, TJs and SJs localize in a tight band at the apicolateral domains of an epithelium, thereby stitching the neighboring cells together. In comparison, the SJs formed between two SSCs are extended along the entire cellular interface. In this way, they seal the enclosure formed by the head-to-head association of the HCC and TCC, which is distinct from the interactions established by these junctions in a monolayer. Due to this unusual arrangement, we call this an atypical septate junction. The junction remained intact and appeared to move from the middle of the elongated cyst towards the base of the NB during the coiling stages due to the coordinated morphogenesis of the HCC and TCC, which is unique to *Drosophila* testis. Previously, TEM analysis of testis sections suggested that the movement of the junction and reshaping of the cyst cells may occur during individualization or early coiling stage (Tokuyasu et al., 1972). Our results obtained from time-lapse imaging of live testis preparations support this hypothesis and provides experimental proof for the repositioning of the junction.

### SJs between the head and tail cyst cells provide mechanical stability to the somatic enclosure during spermatid coiling

Although *PpY-Gal4* is expressed in the SCCs from the post-mitotic stages (Jung et al., 2007), *PpY-Gal4-*mediated expression of *dsDlg1* eliminates the protein from the SCCs only at the last stage of spermatid maturation, when the cyst enters the TE, and leads to premature spermatid release inside the testis. The time-lapse analysis further indicates that these releases occur at an unusual orientation, without the cyst having completely turned, which is not observed in the control testes. This evidence helped to corroborate that the cyst turning is a mechanically stressful event which can only be accomplished if the SCCs are tightly adherent. Loss of SJs may affect the overall integrity of the cyst enclosure, thereby making it vulnerable to physical strain.

Though SJs are classically thought to provide a fluid-access barrier, emerging evidence suggests that they also contribute a mechanical role to the tissue. In HDMEC cell lines, it has been seen that loss of ZO-1 (Dlg1 homolog in mammals) leads to mislocalization of vinculin and a decreased tension on VE-cadherin at the cell–cell junctions (Tornavaca et al., 2015). In MDCK cells, however, knockdown of ZO-1 leads to an increase in tension at cellular junctions, which is caused due to increased afadin and acto-myosin recruitment at the cell cortex. Cell lines with double knockdowns of ZO-1 and afadin are more sensitive to externally applied mechanical stress (Choi et al., 2016). In *Xenopus* embryos as well, loss of ZO-1 leads to an increase in tension on adherens junctions during cytokinesis, leading to cytokinetic defects (Hatte et al., 2018). Very recently, it has been shown that *Drosophila* embryos defective for the SJ protein Kune show an altered mechanical response compared to wild-type counterparts (Carvalho et al., 2018). All these studies collectively suggest that in response to the loss of tight/septate junctions, tension is altered at cell–cell junctions. We hypothesize that in the case of SSCs, loss of SJs might alter the tension felt on adherens junctions. This may have led to cyst disruption and premature sperm release during a mechanically intense event such as cyst rotation. In conclusion, the observations reported here add to this emerging body of data and suggest that apart from serving barrier properties, SJs and TJs help in generating tissue resistance to mechanical strain, which is essential for maintaining organ shape and integrity.

## MATERIALS AND METHODS

### *Drosophila* stocks and culture conditions

All *Drosophila* stocks and crosses were maintained at standard cornmeal *Drosophila* medium at 25°C. Freshly emerged flies are separated from females and were allowed to age for 2–4 days before dissection. For RNAi experiments, the freshly emerged males were kept at 28°C to increase the penetrance of the RNAi. The list of the stocks and their sources are listed in Table S1. We thank the *Drosophila* community for their generous gift of the fly stocks. All experiments involving transgenic Drosophila lines were approved by the Institute Bio-Safety Committee, DBT, Govt of India.

### Fertility assay

Each freshly emerged, *PpY>dsGFP* (control) and *PpY>dsDlg* males were kept with three Canton-S females for 4 days at 28°C to allow for accumulated sperm to be cleared out. On the fourth day, each of these males were extracted and mated with three fresh virgin females (Canton-S) for 24 h at 28°C in separate vials. Then all the flies were discarded. Subsequently, the number of pupae in the vial were counted as a measure of male fertility. Each dot on the plot represents the number of pupae from a single male.

### Immunostaining

For whole mount immunostaining, the testes were dissected in 1× PBS followed by fixation in 4% para-formaldehyde (PFA) for 30 min–1 h at room temperature. Post-fixation, testes were washed with PTX (0.3% Triton-X in PBS), three times for 10 min each. After washing, the samples were incubated with primary antibody diluted in PTX overnight at 4°C. The primary antibody solution was washed off with PTX, and samples were incubated with Alexa-dye tagged secondary antibodies (Invitrogen) for 2–4 h at room temperature. After washing, samples were stained with 0.001% Hoechst 33342 (Sigma-Aldrich), and 10 μM Phalloidin-Atto568/647 (Sigma-Aldrich) for 30 min, washed and mounted in Vectashield^®^ mounting medium (Vector Laboratory Inc.) on a glass slide. For testis squash preparation, the testes were dissected and kept in 50 µl of PBS on a glass slide. A coverslip was placed on top of the sample and gently pressed against the slide. Extra PBS was removed, and the slide was plunged into liquid nitrogen for 2 min. After removing the coverslip, making sure the sample remained on the slide, the slide was then incubated with 95% ethanol, followed by fixation in 4% PFA for 1 h. Further processing was the same as described for whole-mount immunostaining. The primary antibodies used are as follows: Anti-Dlg1 [4F3, DSHB (2/17/11); 1:100], Anti-Cora [C615.16, DSHB (11/23/10); 1:100], Anti-Vasa [DSHB (7/14/16); 1:50], Anti-Alpha-Spectrin [3A9, DSHB (3/26/09); 1:100], Anti-Hts [1B1, DSHB (10/26/03); 1:50], Anti-pH3 [8656-R, Santa Cruz Biotechnology, Inc. (C1513); 1:100], Anti-Eya [eya10H6, DSHB (2/4/10); 1:100] and Anti-Tj (Dorothea Godt, University of Toronto, Canada; 1:1000).

### Transmission electron microscopy

Three-to-four-days-old CantonS flies were dissected in 1× PBS and fixed for 4–6 h, with Karnovsky's fixative (pH 7.4) at room temperature. Samples were washed with 100 mM phosphate buffer (pH 7.4), then post-fixed with K_2_Cr_2_O_7_–OsO_4_ mixture for 2 h on ice, which was followed by 1 h incubation at room temperature. After three to five washes in 100 mM phosphate buffer, the specimens were dehydrated in a graded series of ethanol and propylene oxide. Finally, they were embedded in Durcupan (Fluka, Electron Microscopy Sciences) epoxy resin mix prepared according to the manufacturer’s protocol and polymerized at 60°C overnight. Specimens were then sectioned with a glass and diamond knife on LEICA-EM-UC6 (Leica Microsystems). Ultrathin sections were collected on Formvar-carbon coated copper slots. These sections were examined on Libra120EFTEM Transmission Electron microscope (Carl Zeiss AG).

### Image analysis and quantification

Images were acquired using Olympus FV1000SPD and FV3000SPD Laser scanning confocal microscopes (Olympus Co., Japan). Live Imaging was performed on FV1000SPD, as described by (Dubey et al., 2016). The images were analyzed using Fiji- ImageJ (http://fiji.sc/Fiji). The pair-wise significance of difference (*P*-value) was estimated using the Mann–Whitney *U*-test. ‘*n*’ represents the number of testes analyzed.

## Supplementary Material

Supplementary information
