# Supplementary material for: Atypical septate junctions maintain the somatic enclosure around maturing spermatids and prevent premature sperm release in Drosophila testis
Source: Biol Open. 2019 Jan 11;8(2):bio036939. doi: 10.1242/bio.036939 (PMC6398457; doi:10.1242/bio.036939)
Supplement: Supplementary information [file biolopen-8-036939-s1.pdf]

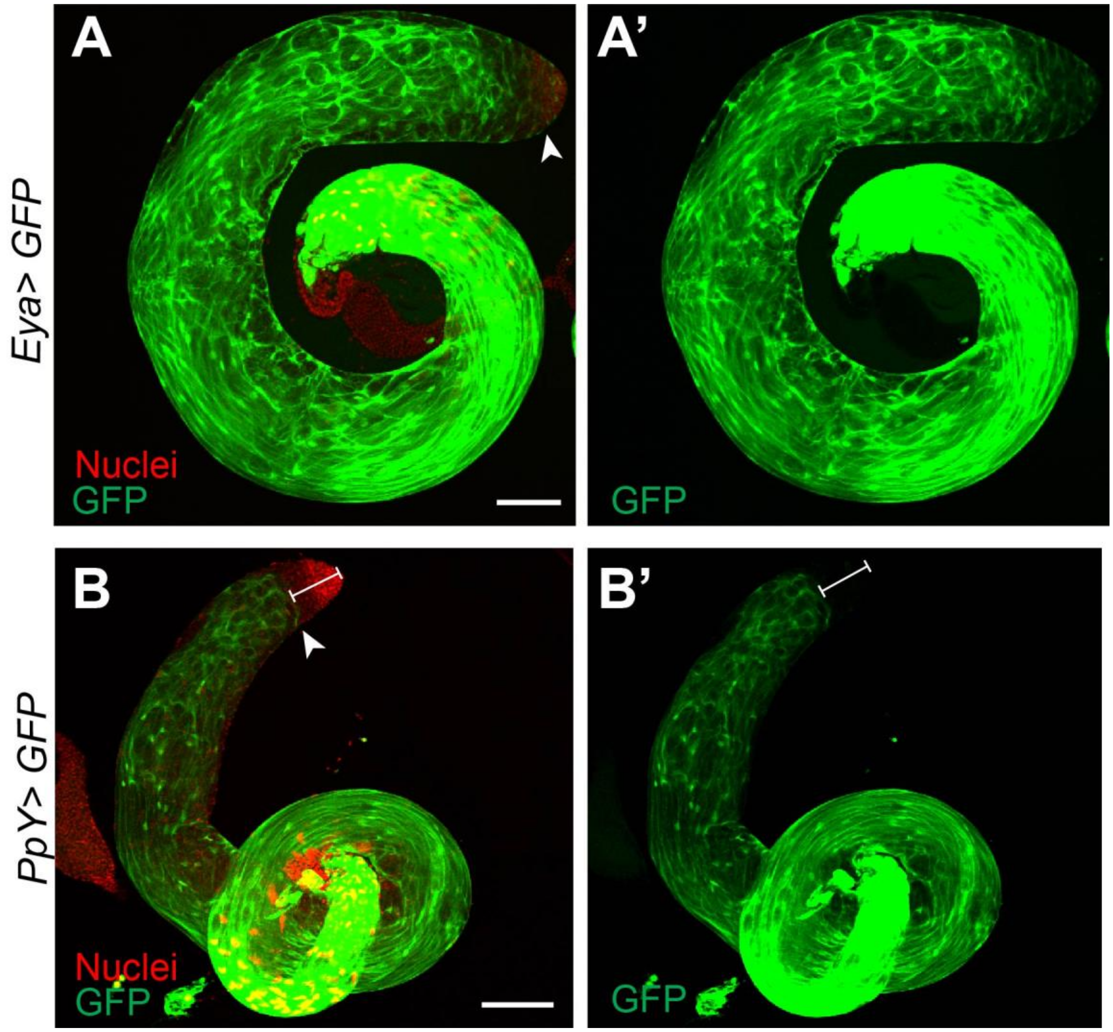

Fig S1. Expression pattern of *eya-Gal4* and *PpY-Gal4.*

A-A') Hoechst staining (red) in *eya>GFP* (*eya-Gal4/UAS-GFP*) testes shows that eya expression pattern begins from spermatogonial stages (arrowhead)

B-B') Hoechst staining (red) in *PpY>GFP* (*UAS-GFP/+; PpY-Gal4/+*) testes reveals that the onset of PpY expression (arrowhead) is from the meiotic stages. Double-sided blunt arrow marks the stages which do not express the Gal4.
(Scale- 50μm)

Biology Open • Supplementary information

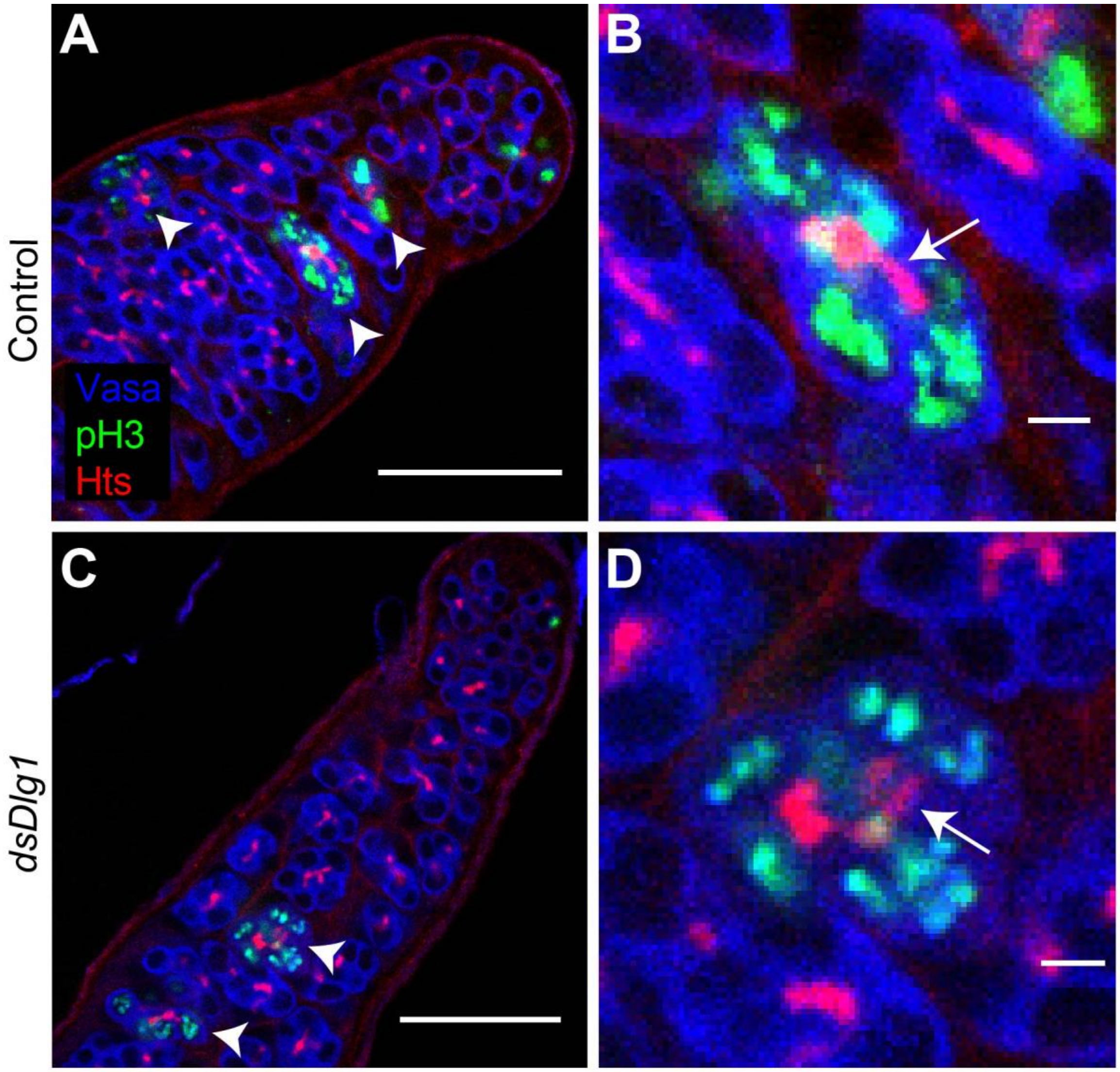

Fig S2. Somatic knockdown of Dlg1 does not alter the fusome morphology in spermatogonial cysts.

The *eya-Gal4/+* (control) (n=10) and *eya>dsDlg1* (n=13) testes were stained with anti-Vasa (blue), anti-pH3 (green) and anti-Hts (red) marking the germline cells, mitotic clusters and fusome, respectively.

Arrowheads in **A** and **C** mark mitotic cysts in which germ cells are positive for pH3 (Scale-50 μm). High magnification images in **B** and **D** indicate the branched fusomes (arrowheads) marked by Hts (Scale-5 μm).

Biology Open • Supplementary information

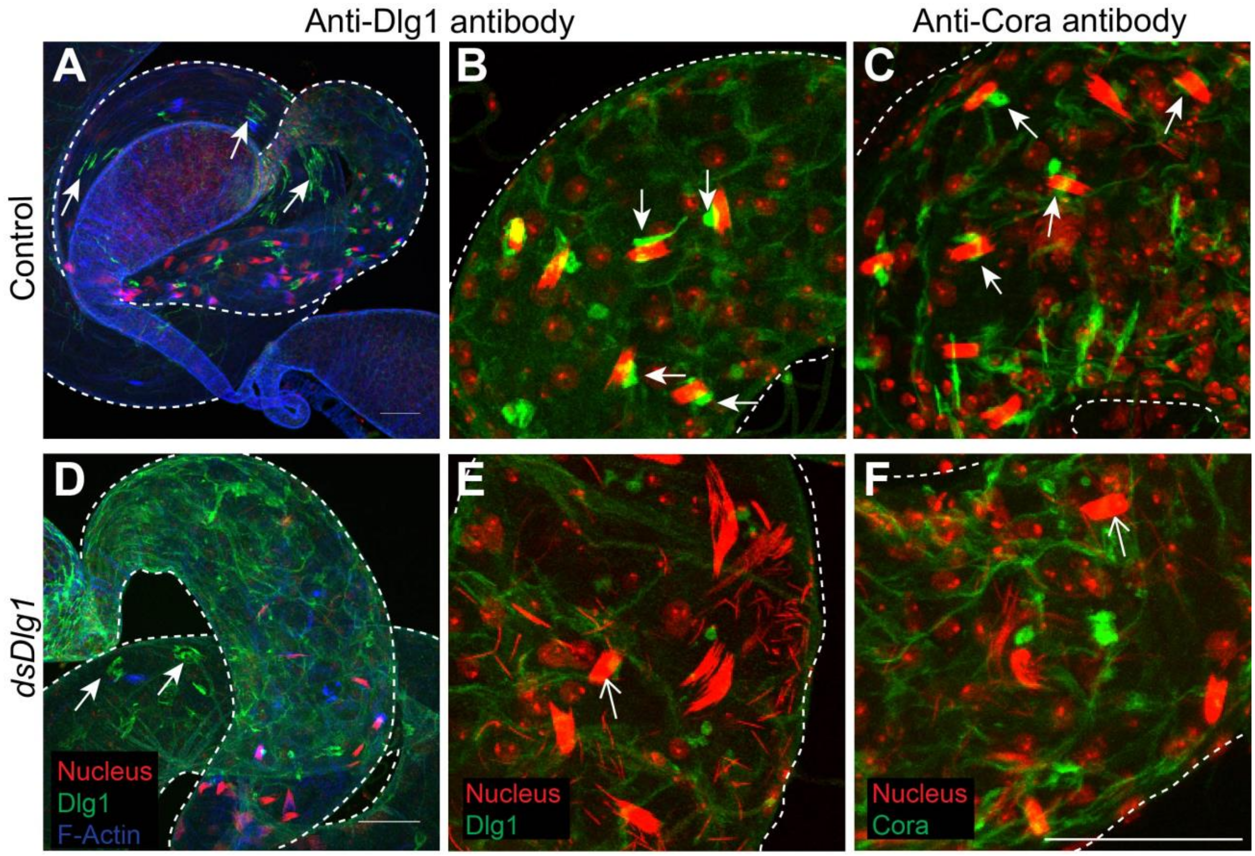

Fig S3. Validation of the stage-specific Dlg1 knockdown in SCCs region.

Control and *PpY>dsDlg1* testes were stained with Hoechst (red), Phalloidin (blue) and anti-Dlg 1 antibody (green; **A, B, D, E**) or anti-Cora antibody (green; **C** & **F**).

A-C) Filled arrows indicate the presence of an intact junction, as marked by anti-Dlg1 antibody during elongation/individualization stages **(A)**, and coiled stages **(B)**, or anti-Cora antibody during coiled stages **(C)**.

D-F) Filled arrows indicate the presence of an intact junction, as marked by the anti-Dlg1 antibody, during elongation/ individualization stages **(D)**. However, Dlg1 **(E)** and Cora **(F)** are lost from around the spermatid head bundle in the coiled stages in the TE (thin arrows) upon knockdown of Dlg1.

(Scale-50 μm)

Biology Open • Supplementary information

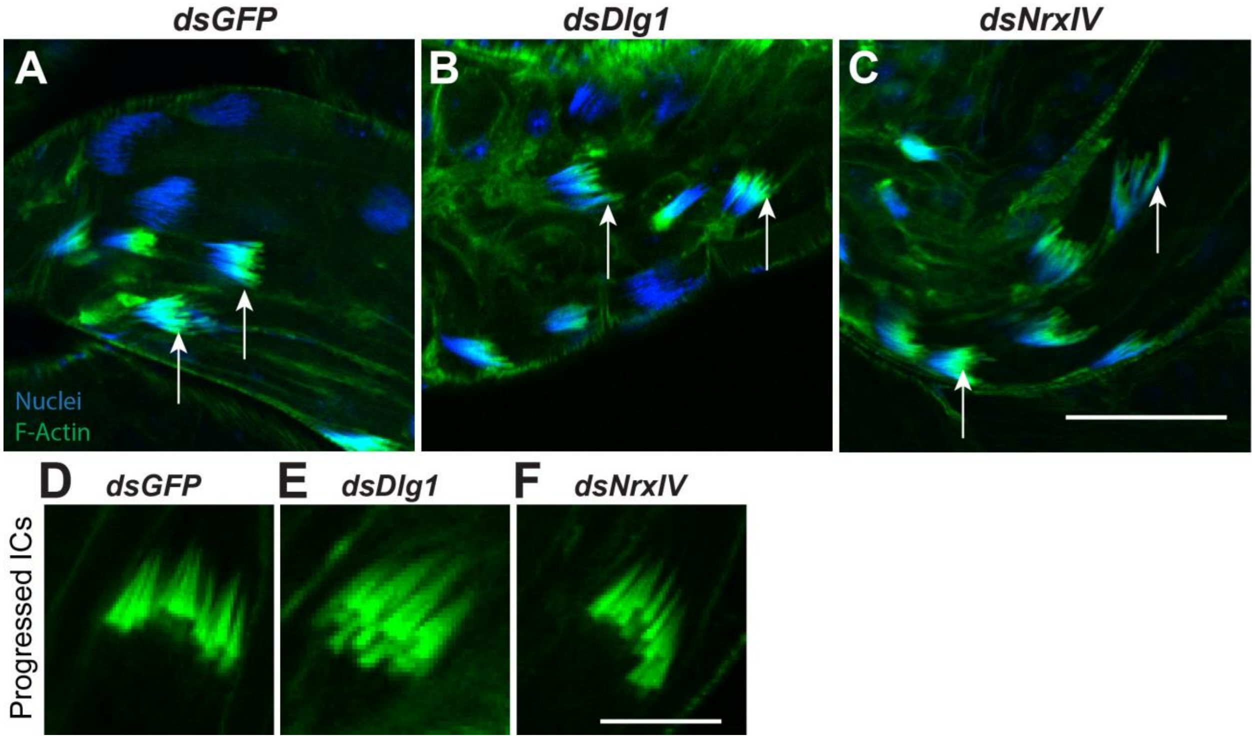

Fig S4. Individualization is not affected by the loss of Dlg1 in SCCs at the late stages.

A-C) Hoechst (blue) and Phalloidin (green) stained testes of *PpY>dsGFP* (control) **(A)**, *PpY>dsDlg1* ***(B)*** and *PpY>dsNrxIV* ***(C)***. Arrows mark early individualization cones.

D-F) Phalloidin stained progressed ICs in the control **(D)**, *PpY>dsDlg1* **(E)** and *PpY>dsNrxIV* **(F)** testes.
(Scale-50 μm)

Table S1. List of Fly stocks used in the study

Stock	Source
Nrg-GFP[PT]	Bloomington Drosophila Stock Center (BDSC)- BL-6844
NrxIV-GFP[PT]	BDSC; BL-50798
Lac-GFP[PT]	BDSC; BL-6833
ATPα-GFP[PT]	BDSC; BL-6834
Nrv2-GFP[PT]	BDSC; BL-6828
Dlg-GFP[PT]	BDSC; BL-50859
eyaA3-Gal4	Kindly provided by Prof. Benny Shilo, Weizmann Institute of Science, Israel (Rotkopf et al., 2011).
PpY-Gal4	Kindly provided by Prof. Benny Shilo, Weizmann Institute of Science, Israel (Rotkopf et al., 2011).
UAS-eGFP	BDSC; BL-5431
UAS-dsDlg1	BDSC; BL-25780
UAS-dsNrxIV	Vienna Drosophila Resource Centre (VDRC); GD-8353
UAS-dsGFP	BDSC; BL-9330
Protamine A-eGFP (ProtA-GFP)	Prof. John Belote, Syracuse University, USA
Protamine B-dsRed (ProtB-dsRed)	Prof. John Belote, Syracuse University, USA
UAS-mCD8-RFP	BDSC; BL-27398
UAS-Dicer	VDRC; GD-60008

Reference-

Rotkopf, S., Hamberg, Y., Aigaki, T., Snapper, S.B., Shilo, B., and Schejter, E.D. (2011). The WASp-based actin polymerization machinery is required in somatic support cells for spermatid maturation and release. *Development*. **2739**, 2729–2739.

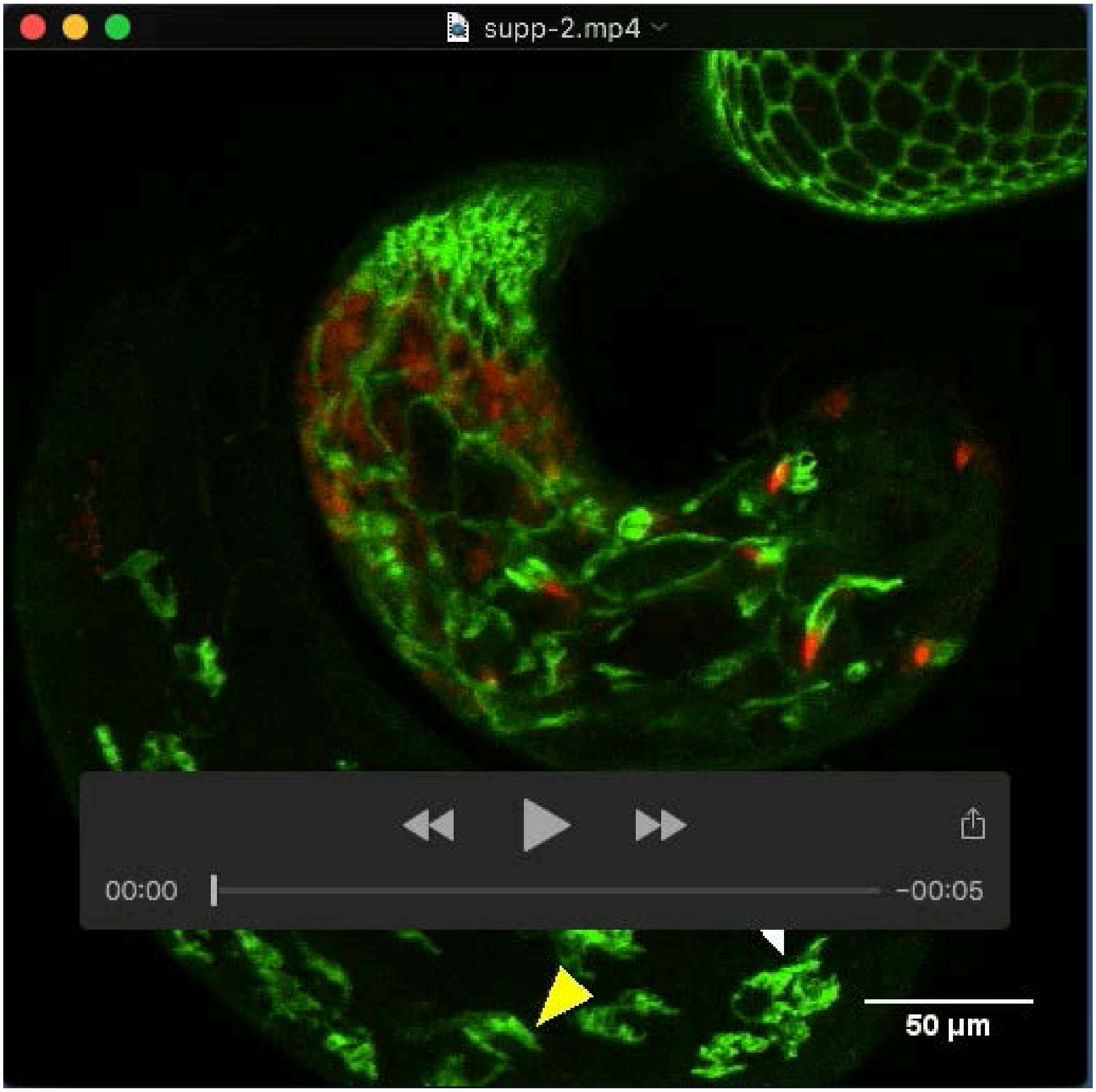

Movie 1. Movement of Nrg-GFP from the middle region of the testis towards the base: Time-lapse images of an *Nrg-GFP*^{PT}*/Y; ProtB-dsRed/+* testis showing the movements of SJs (yellow and white arrowheads) marked by Nrg-GFP towards the testis base.

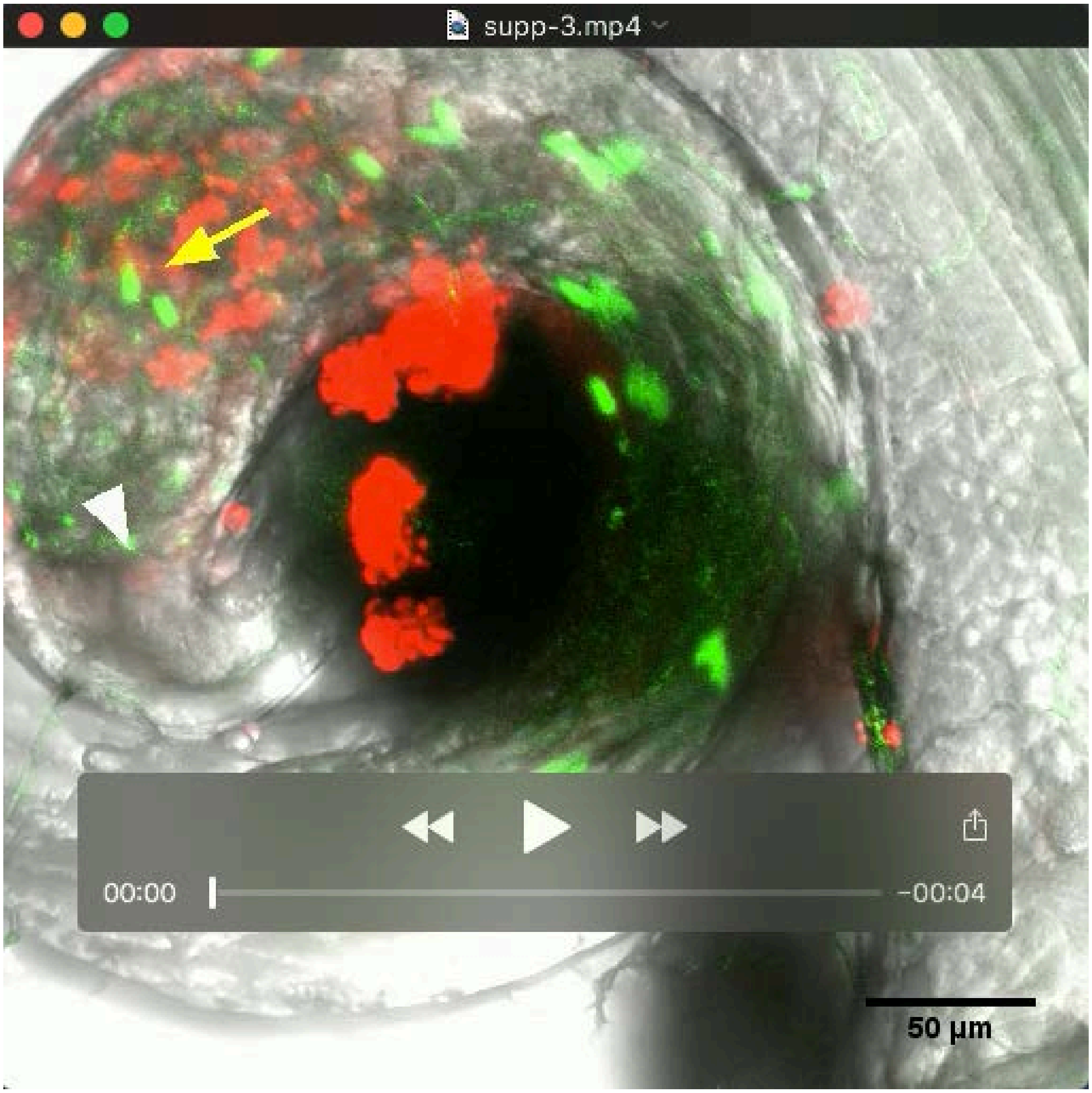

Movie 2. Spermatids exit in the direction of SV in Control testis: Time-lapse images of a *ProtA-GFP/UAS-Dicer; PpY-Gal4>UAS-mCD8-RFP/+* (Control) testis depict retraction of an NB (green; arrow) from the HCC (red) towards the SV (indicated by arrowhead).

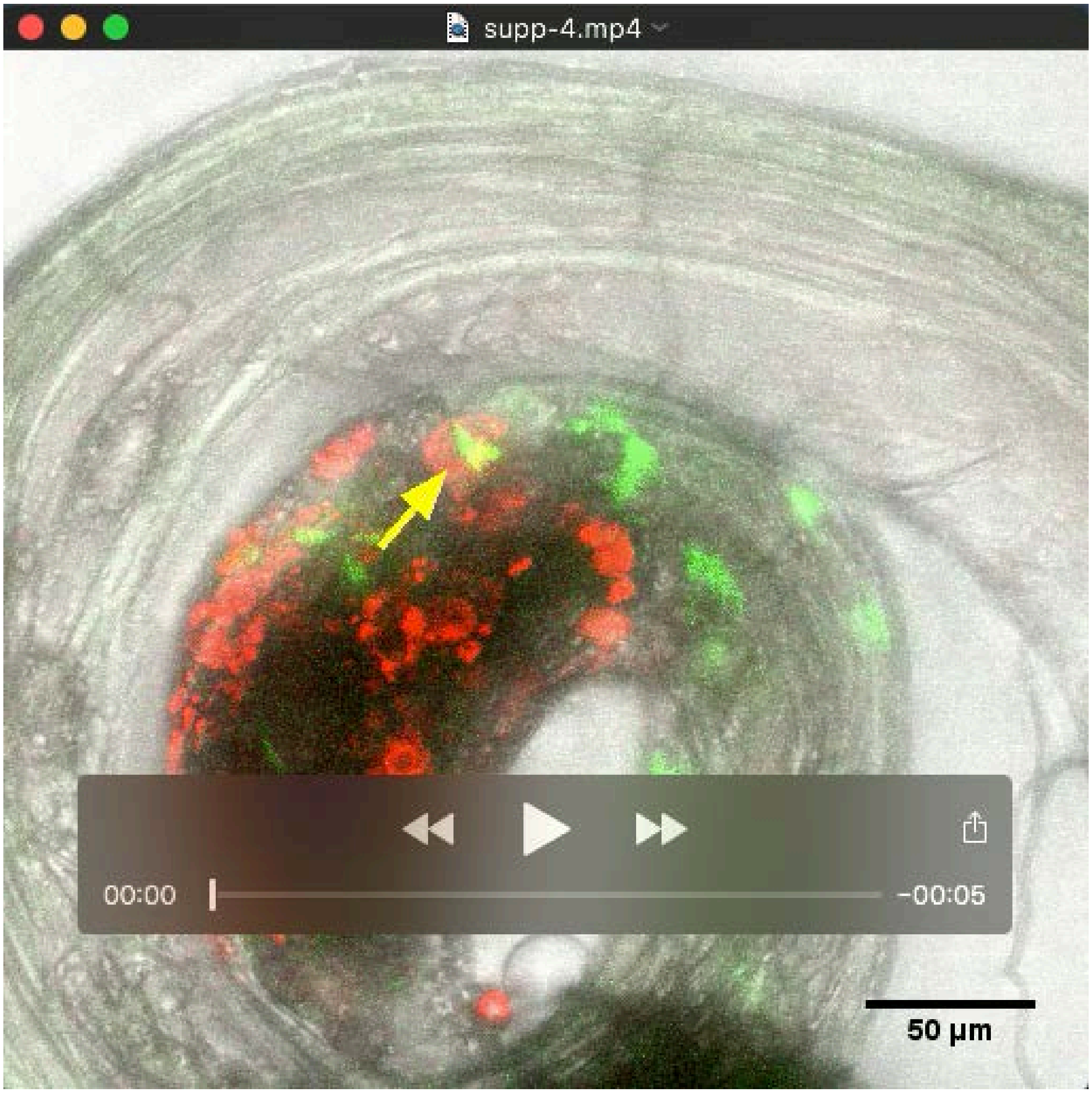

Movie 3. Spermatids are released within the TE in Dlg1 knockdown testis: Time-lapse images of a *ProtA-GFP/UAS-dsDlg1; PpY-Gal4>UAS-mCD8-RFP/+* depict retraction of an NB (green; arrow) from the HCC (red) even though the NB is not oriented away from the SV (arrowhead). As a result, the spermatids are released inside the testis.
